# 3-Aminopropylsilatrane and Its Derivatives: A Variety of Applications

**DOI:** 10.3390/molecules27113549

**Published:** 2022-05-31

**Authors:** Sergey N. Adamovich, Elizaveta N. Oborina, Arailym M. Nalibayeva, Igor B. Rozentsveig

**Affiliations:** 1 A.E. Favorsky Irkutsk Institute of Chemistry, Siberian Branch of the Russian Academy of Sciences, 1 Favorsky Street, 664033 Irkutsk, Russia; oborina@irioch.irk.ru (E.N.O.); i_roz@irioch.irk.ru (I.B.R.); 2 D.V. Sokolskiy Institute of Fuel, Catalysis and Electrochemistry, 142 Kunaev Street, Almaty 050010, Kazakhstan; aray77@mail.ru

**Keywords:** silatranes, 3-aminopropylsilatrane, hybridization, functionalization, bioactivity, surfaces, coatings, materials, application

## Abstract

Silatranes arouse much research interest owing to their unique structure, unusual physical–chemical properties, and diverse biological activity. The application of some silatranes and their analogues has been discussed in several works. Meanwhile, a comprehensive review of the wide practical usage of silatranes is still absent in the literature. The ability of silatranes to mildly control hydrolysis allows them to form extremely stable and smooth siloxane monolayers almost on any surface. The high physiological activity of silatranes makes them prospective drug candidates. In the present review, based on the results of numerous previous studies, using the commercially available 3-aminopropylsilatrane and its hybrid derivatives, we have demonstrated the high potential of 1-organylsilatranes in various fields, including chemistry, biology, pharmaceuticals, medicine, agriculture, and industry. For example, these compounds can be employed as plant growth biostimulants, drugs, optical, catalytic, sorption, and special polymeric materials, as well as modern high-tech devices.

## 1. Introduction

**Scheme 1:**
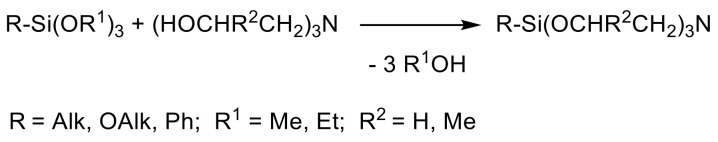
Synthesis of silatranes.

Atranes are well-known and very interesting elemental/organometallic derivatives of amino alcohols, in particular trialkanolamines (see Refs. [1,2,3,4,5,6] and literature cited therein). Historically, the first atranes were «silatranes», which were synthesized by the transesterification of trialkoxysilanes with triethanolamine or triisopropanolamine (Scheme 1) [7,8].

**Figure 1:**
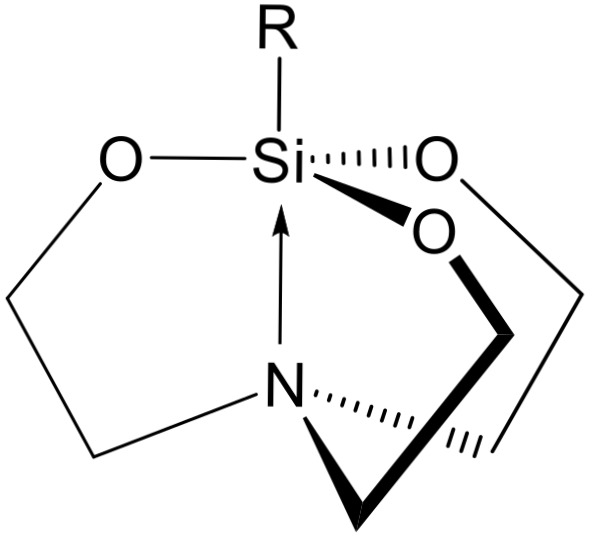
Chemical structures of silatranes.

Various studies have demonstrated that silatrane molecules contain the so-called intramolecular coordinate bond (N→Si), which imparts them a unique tricyclic structure (Figure 1; see Refs. [9,10,11,12] and literature cited therein).

**Scheme 2:**
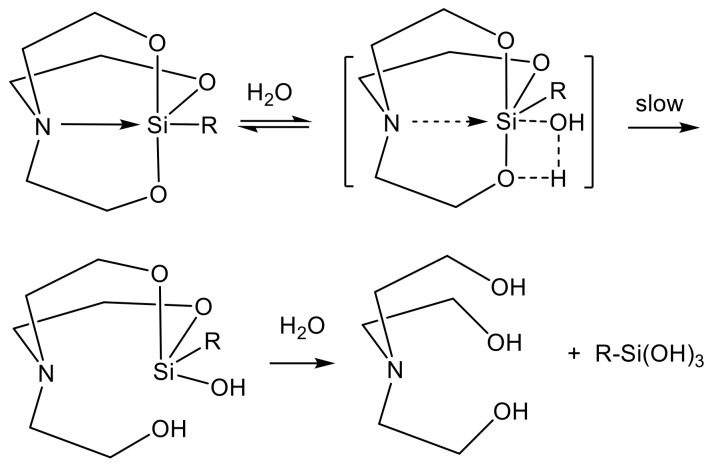
Hydrolysis of silatranes.

Such a skeleton of silatranes with hypervalent (pentacoordinated) silicon predetermines their unusual physical and chemical properties, for example, a strong dipole moment of the molecule, a strong electron-donor effect of the silatrane group, and, hence, a high reactivity [9,10,11,12,13,14,15,16,17,18,19]. In contrast to the starting alkoxysilanes, a striking feature of silatranes is their relative resistance to hydrolysis (Scheme 2) [20,21,22,23].

In recent years, this property of silatranes has been employed in a new straightforward strategy for the preparation, modification, and functionalization of surfaces that find diverse applications. It has been shown that silatrane coatings, in contrast to alkoxysilane ones, ensure a more facile formation of thin and uniform films, as well as better molecular regularity and availability of the functional groups on the various surfaces (glass, mica, paper, cotton, natural and synthetic polymers, silica gels, metal oxides) (see Refs. [24,25,26,27,28] and literature cited therein).

**Scheme 3:**
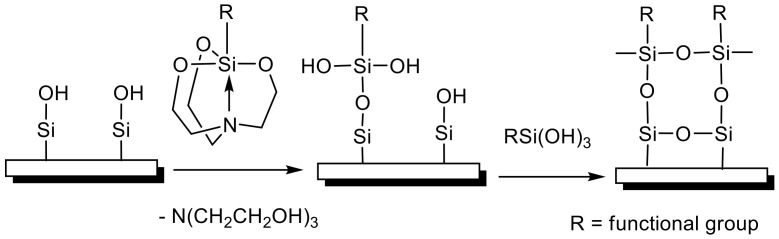
Modification of silica gel with functional silatranes.

The controlled hydrolysis of silatranes allows the mild silanization of surfaces of, e.g., silica gels. The surface modification of silica gel is the result of an interaction between silatrane and hydroxyl groups of silica gel and involves three stages:

(a) The silatrane hydrolysis leading to the release of triethanolamine to afford silanol R-Si(OH)_3_;
(b) Silanol condensation with the hydroxyl group of silica gel to generate a silanol intermediate;
(c) Condensation of the second silanol molecule with both the hydroxyl group of the intermediate and the OH group of silica gel to form the –Si–O–Si– chains. As a result, a uniform self-assembled functional siloxane monolayer is formed (Scheme 3) [24,25,26,27,28].

Finally, the discovery of M.G. Voronkov made a real sensation. In 1963, he showed that, unlike biologically inert silanes, siloxanes and silicones, silatranes exhibit a rich and diverse biochemical, physiological, and pharmacological activity [9,10,11,12,29,30,31,32].

Further on, the biological effect of silatranes became the object of in-depth studies in Russia, the USA, Poland, Germany, China, India, and other countries. It has been found that some silatranes stimulate the biosynthesis of proteins and nucleic acids, regulate the activity of enzymes and hormones, and exert a protective effect during hypo- and hyperthermia, oxygen starvation, and radiation damage [29,30,31,32]. Without having a direct action, these compounds exhibit anticoagulant, hypocholesterolemic, and antitumor effects. Thus, the unique physical, chemical, and biological properties of silatranes can, and, in some cases, already ensure, their application in medicine, cosmetology, animal husbandry, poultry farming, practical etymology, and crop production [9,10,11,12,29,30,31,32].

Molecular hybridization (MH) is an approach to the expedient development of new substances (for example, drugs) when chemical compounds are obtained by the combination of two or more pharmacophoric units into one molecule. MH implies not only an additive but also a synergistic effect of pharmacophoric components. A principle of MH, “two heads are better than one”, has been successfully used for the synthesis of, for example, new hybrid compounds with enhanced anticancer, anti-inflammatory, and anti-microbial properties (see Refs. [33,34,35,36,37,38,39] and literature cited therein).

As experience shows, the silatrane-based MH strategy can be applied not only for the design of improved drugs but also for the preparation of multitargeting special materials.

**Figure 2:**
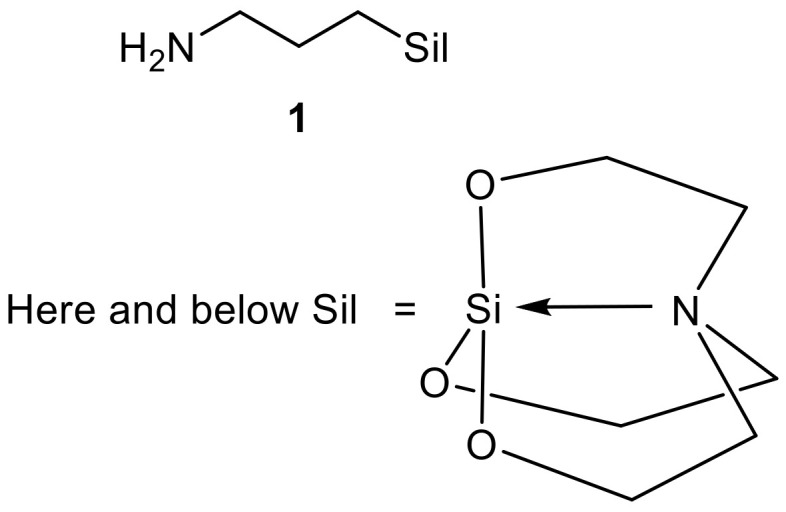
Chemical structures of 3-Aminopropylsilatrane (APS) (**1**).

To date, numerous silatranes with various functional substituents at the silicon atom have been synthesized and investigated. Among these compounds is commercially available 3-aminopropylsilatrane (APS), H_2_N(CH_2_)_3_-Si(OCH_2_CH_2_)_3_N (**1**) (Figure 2) [40].

In the present review, based on the results of numerous previous studies, using the commercially available silatrane APS (**1**) and its hybrid derivatives (**2**–**46**) as an example, we have demonstrated a wide range and high potential of the practical application of silatranes in general.

## 2. Silatranyl Physiologically Active Compounds

### 2.1. Silatranyl Plant Growth Regulators

It was previously established that the treatment of seeds of barley, potatoes, carrots, radishes, and tomatoes with aqueous solutions of 1-methyl-, 1-chloromethyl-, and 1-ethoxysilatranes enabled to increase the yield of these agricultural crops and the resistance of plants to unfavorable environmental effects [29,30,31,32].

At the same time, 3-aminopropylsilatrane (APS) has generated a wide interest due to its plant-growth-regulating activity and protective properties.

**Figure 3:**
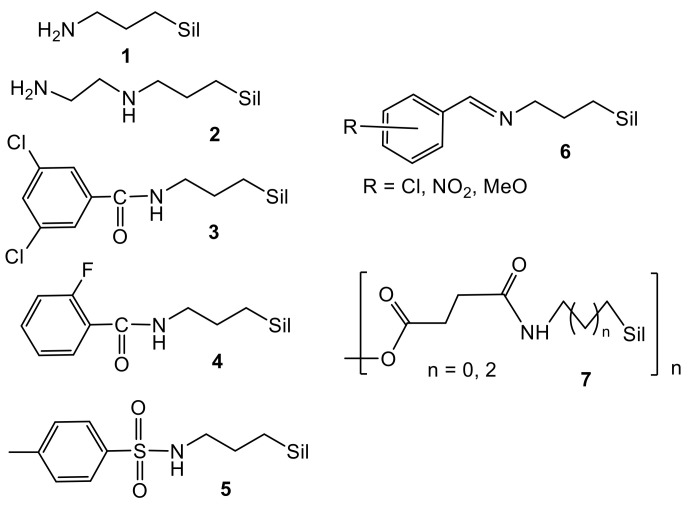
Chemical structures of silatranyl plant growth regulators (**1**–**7**).

The stimulating effects of silatranes (**1**,**2**) (Figure 3) were reported on the early growth of wheat and maize seedlings. These silatranes showed a favorable effect on the growth with respect to control [41].

Aminopropylsilatrane derivatives (**3**–**5**) (Figure 3) demonstrated an excellent activity in the regulation of the stem and root growth of corn and radish. For example, the germination efficacies for APS and compounds (**3**,**4)** were 60% and 200%, respectively [42]. Moreover, the seed germination test revealed that the compounds (**1**–**5**) possessed better activity than 1-chloromethylsilatrane and 1-chloropropylsilatrane and their effect was highest in the micromolar concentration ranges (10^−7^ mol/L).

Silatranes (**6**) (Figure 3) were tested for their plant growth-regulating activity. The compounds showed good results in the regulation of the root/stem growth of turnips and corn at a concentration of 40 ppm. Furthermore, their activity was stronger than that of the well-known plant hormone brassinolide (125 and 72 %, respectively) [43].

The results obtained by Istratov et al. indicated the possible creation of biodegradable and biocompatible amphiphilic polymers bearing the silatrane groups. It was found that APS-containing polymers (**7**) (Figure 3) positively influence the germination of wheat, oat, and rye seeds (germination percentage, H_2_O—77%, germination percentage, (**7**)—98%) [44].

Thus, it was found that silatrane-containing compounds act as biostimulants. This increases not only the yield but also the resistance of plants to adverse environmental factors.

### 2.2. Silatranyl Antifungal Agents

**Figure 4:**
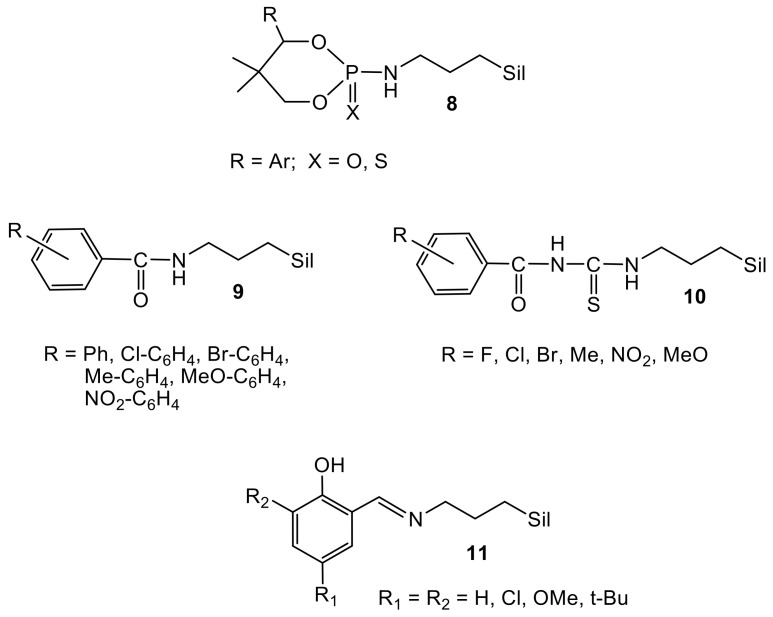
Chemical structures of silatranyl antifungal agents (**8**–**11**).

Some dioxaphosphinanes are known to exhibit good antifungal activity. Wan et al. synthesized hybrid phosphoryl-aminopropylsilatranes (**8**) (Figure 4) and investigated their biological activity.

The results confirmed that the fungicidal activity of the obtained silatranes exceeded that of the parent compounds. For instance, at a concentration of 50 mg/L, silatranes (**8**) inhibited necrotrophic fungus *Botrytis cinerepers* and pathogen fungus *Bipilaris maydis* by 87% and 85%, respectively [45].

The substituted aminopropylsilatranes (**9**) (Figure 4) were synthesized [46]. The antifungal activity of the compounds (**9**) was preliminarily evaluated on *Fusarium oxysporium, Rhizatonia solani, Gibberella zeae, Biopolaris mayalis*, and *Dochiorella gregaria*. It was established that silatranes (**9**) had remarkable activity against all the fungi (70–80%), especially *Fusarium oxysporium* and *Rhizatonia solani* (up to 90%).

Ten substituted benzoyl aminopropylsilatranes (**10**) (Figure 4) were synthesized by the reaction of APS with various substituted benzoyl chlorides [47]. The antifungal activity was tested for compounds (**10**) on *Cotton Fusarium Wilt, Rice Sheath Blight, Cucumber Mildew, Wheat Scab, Apple Ring Rot,* and *Corn Leaf Blight* (the microconcentration was 50 μ1/L). It was found that the compounds exhibited excellent activity with respect to all the fungal diseases (60–80%), especially *Cucumber Mildew* (up to 99%).

Schiff-base-type silatranes (**11**) (Figure 4) were obtained from APS and three derivatives of salicylaldehyde with 3,5-dichloro-, 3-methoxy-, and 3,5-di-tert-butyl- substituents [48]. The antifungal activity of these compounds was evaluated using three species of fungi (*Aspergillus fumigatus, Penicillium chrysogenum, Fusarium*). The MIC values (µg/mL) for compounds (**11**) were compared with those of the standard ones: Capsofungin (MIC = 0.3 µg/mL) and Kanamycin (MIC = 4 µg/mL). The silatranes with chloro and *t*-Bu substituents exerted no effect against fungi (MIC > 128 µg/mL), while silatranes with the methoxy substituent at the aromatic ring showed very good antifungal activity (MIC = 0.08 µg/mL).

The above examples indicate that the introduction of the silatrane fragments to biological molecules sharply enhances their antifungal activity.

### 2.3. Silatranyl Antimicrobial Agents

**Figure 5:**
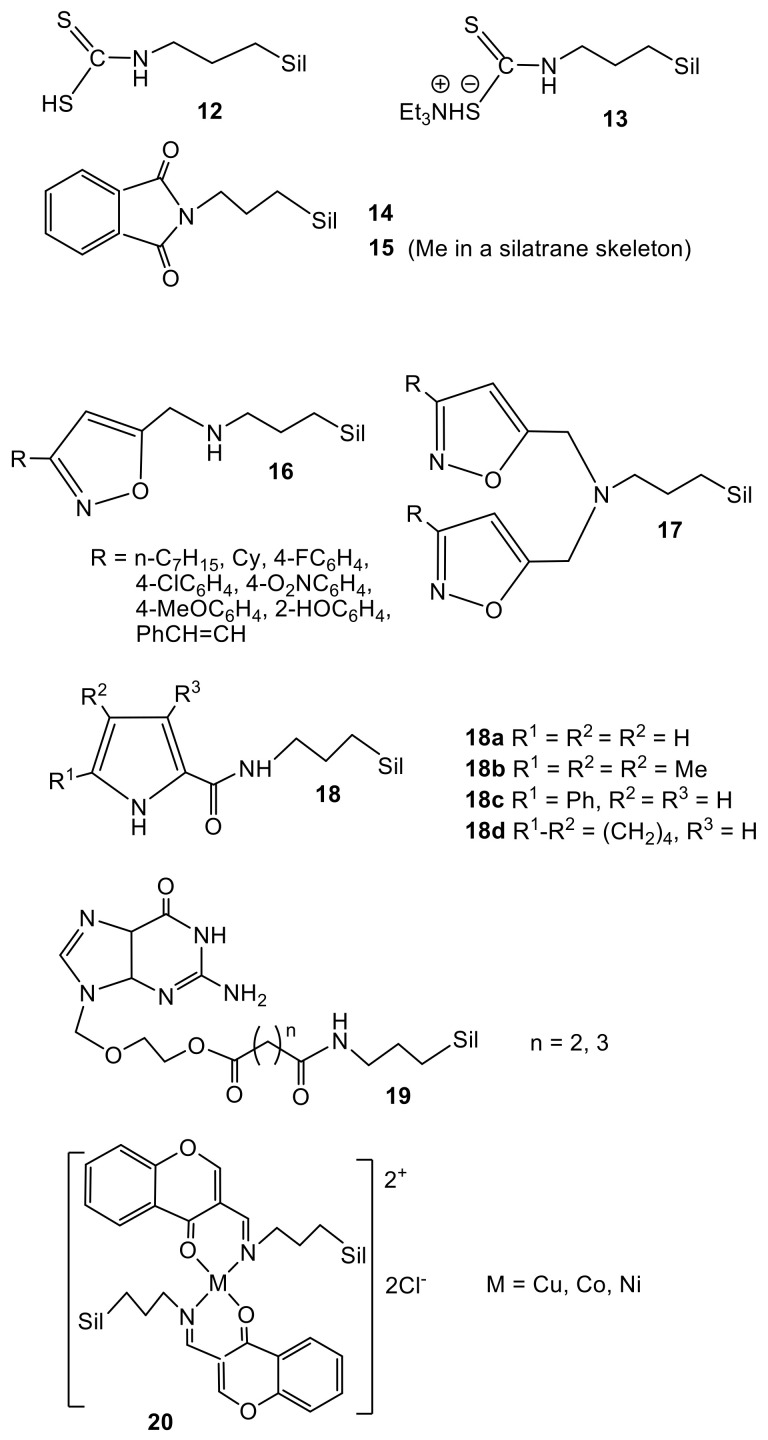
Chemical structures of silatranyl antimicrobial agents (**12**–**20**).

Microbes can cause various pathologies, from mild infections to deadly diseases. It was disclosed [49] that APS (**1**) reacted with CS_2_ to form 3-siltranylpropyldithiocarbamic acid (**12**), which can provide triethylammonium-3-silatranyl-propyldithiocarbamate (**13**) (Figure 5). 

The antimicrobial activity of the compounds (**12**,**13**) was evaluated against bacterial culture of *Bacillus subtillus*, *Escherichia coli*, and *Staphylococcus aureus*. The results obtained indicate that silatrane (**13**) is very active toward the growth inhibition of bacteria (MIC 1.80 mg/mL).

Silatranes (**14**) and (**15**) (Figure 5) containing the phthalimide group were prepared by the transesterification of N-(triethoxysilylpropyl)phthalimide with triethanolamine and trisisopropanolamine, respectively [50]. Compounds (**14**) and (**15**) were tested with respect to bacterial strains of Gram-positive *Staphylococcus aureus* and Gram-negative *Acinetobacter baumannii*, *Pseudomonas aeruginosa,* and *Escherichia coli*. Both silatranes were found to be effective antimicrobial agents against all microorganisms tested. The antibacterial screening pointed out that compounds **14** and **15** were equipotent for both Gram-positive and Gram-negative bacteria (MIC 0.20 mg/mL).

A series of novel organic–inorganic hybrids, combining one (**16**) or two (**17**) units of the isoxazole motif and one silatrane unit in a single molecule, were synthesized (Figure 5) [51]. The screening of silatranes (**16**) and (**17**) for antimicrobial activity against *Enterococcus durans*, *Bacillus subtilis*, *Escherichia coli*, and *Pseudomonas aeruginosa* revealed that all the test samples were active only against Gram-positive microorganisms. The silatrane R = MeOC_6_H_4_ displayed minimal inhibitory concentration (MIC 12.5 and 6.2 μg/mL) against *E. durans* and *B. subtilis* as compared with standard drug Gentamicin (MIC 25 and 50 μg/mL). Overall, the results obtained confirm that the combination of two, three, or more biologically active species in one molecule can lead to a synergetic effect of their activity.

APS (**1**) and 2-(trichloroacetyl)pyrroles were used as precursors to obtain hitherto unknown 1*H*-R-pyrrole-2-carboxamidesilatrane hybrids (**18**) (Figure 5) [52]. The starting silatrane (**1**) and pyrrolcarboxamide-linked silatranes (**18a**–**d**) were checked in vitro for antimicrobial activity against bacterial strains of Gram-positive *Enterococcus durans*, *Bacillus subtilis,* and Gram-negative *Escherichia coli*. It was established that pyrrole-silatranes (**18**) exhibited moderate-to-excellent activity (MIC, 62.5–125 μg/mL) with respect to Gram-positive microorganisms. Meanwhile, tetrahydroindole-silatranes (**18d**) was effective against Gram-negative *E. coli*. The MICs of silatranes (**18b**–**d**) were by two times lower for *E. durans* and *B. subtilis* and by 8 to 16 times lower for *E. coli* as compared with the starting silatrane (**1**). Maximum antibacterial activity (MIC, 3.1 and 6.2 μg/mL) against *E. durans* and *B. subtilis* was demonstrated by silatrane (**18a**) as compared with standard aminoglycoside antibiotic Gentamicin (MIC 25 and 50 μg/mL).

Hepatitis B virus (HBV) infection causes serious problems for human health (cirrhosis or liver cancer and even death). Acyclovir (ACV) is used to inhibit herpes virus but is not efficient against HBV. Therefore, APS/acyclovir hybrids were synthesized (**19**) (Figure 5) [53,54]. The compounds (**19**) were found to be effective inhibitors of HBV both in vitro and in vivo (for ACV, IC_50_ > 1000 μM; for (**19**), IC_50_ 35–160 μM).

A new series of Cu(II), Ni(II), and Co(II) complexes (**20**) were obtained from 3-formylchromoniminopropylsilatrane (Figure 5) [55]. The biological activity of the ligand and metal complexes was studied on *Klebsiella pneumoniae*, *Staphylococcus aureus*, *Escherichia Coli*, and *Bacillus subtilis* by the well diffusion method. The zone of inhibition values were measured at 37 °C for 24 h. Antimicrobial screening tests showed better results for the metal complexes than for the ligand.

Thus, both microbes and viruses were found to be susceptible to APS-containing compounds. The combination of silatranyl and bio-functional fragments significantly increases the antimicrobial and antiviral activity.

### 2.4. Silatranyl Antiparasitic Agents

Parasites are the primary cause of many diseases of humans, animals, and plants. Among such disorders, giardiasis is one of the most grave ones, distressing millions of people, especially children. Another serious parasite-induced pathology is trichomoniasis. These diseases are posing a serious threat to millions of men and women every year.

**Figure 6:**
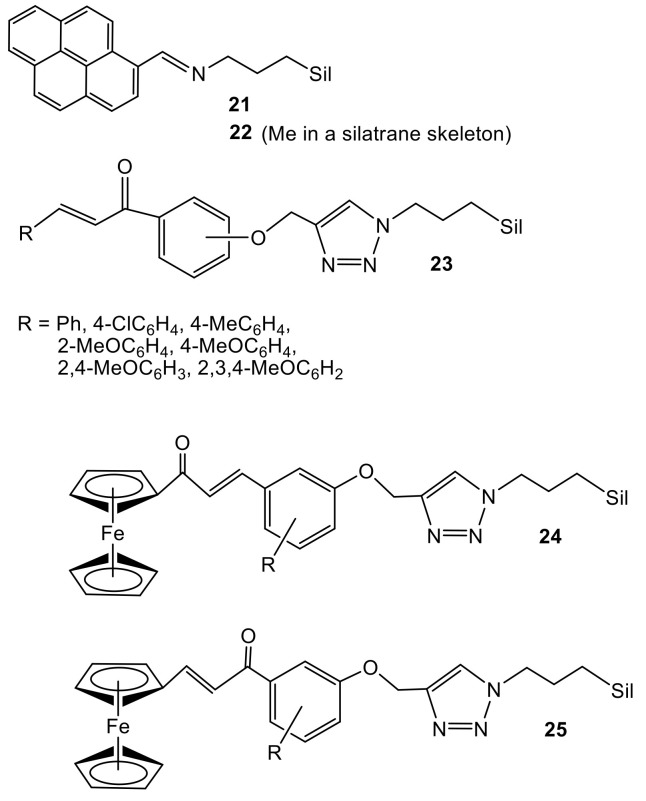
Chemical structures of silatranyl antiparasitic agents (**21**–**25**).

Therefore, a great deal of effort was invested in finding efficient antiparasitic agents. For example, Singh et al. prepared two-component pyrene–silatrane hybrids (**21**, **22**) by the reaction of pyrene-carboxaldehyde and APS followed by transesterification of the condensation product using triethanolanine or triisopropanolamine (Figure 6).

The studies showed that both compounds were capable to act against *Giardia lamblia* and *Trichomonas vaginalis*. Moreover, silatrane (**22**) had a better effect on *T. vaginalis* than the standard drugs, for example, Metronidazole IC_50_ 10 μM; silatrane (**22**) IC_50_ 1.75 μM [56].

A series of three-component (chalcone + triazole + silatrane) hybrids (**23**) were synthesized using a facile synthetic route (Figure 6). The blend of three different pharmacologically active moieties into a single scaffold resulted in a synergistic effect in their bio-activity. The antiparasitic activity of the compounds (**23**) was evaluated against parasites (*G. lamblia* and *T. vaginalis*) in comparison to the standard drug. All the compounds displayed significant activity against the parasites with half-maximal inhibitory concentration (IC_50_) values ranging from 18.24 to 50.21 μM. The entire library of compounds was found to be more active than metronidazole (55.85–62.48 μM) [57].

Moreover, four-component (ferrocene + chalcone + triazole + silatrane) hybrids (**24**,**25**) were produced with the aim of the association of the pharmacological activity of the constituting moieties into a single molecular structure (Figure 6). The compounds (**24**,**25**) were evaluated for their biological activity against parasite strains [58]. These organosilatranes exhibited excellent efficacy against *G. lamblia* and *T. vaginalis*. Silatranes **24** and **25** (where R = o-Me) were the most promising candidates displaying the phenomenal activity against *G. lamblia* (IC_50_ 0.57 and 1.57 µM, respectively).

The performed investigations [56,57,58] demonstrate the importance of the synthesis and application of APS-containing multicomponent antiparasitic agents.

### 2.5. Silatranyl Anticancer Agents

Way back in the 1970s, Voronkov et al., began to search for new anticancer agents among silatranes. Eventually, it was found that the introduction of some silatranes (including APS and its derivatives) into the organism increased its resistance to the development of malignancies [29,30,31,32]. Later, it was established that APS enhanced the antitumor activity of the cyclo-phosphamide drug [59]. The 3-(arylideneamino)propylsilatranes and their metal complexes inhibited the growth of Erlich ascites cancer in mice by 41 to 53% [60,61].

**Figure 7:**
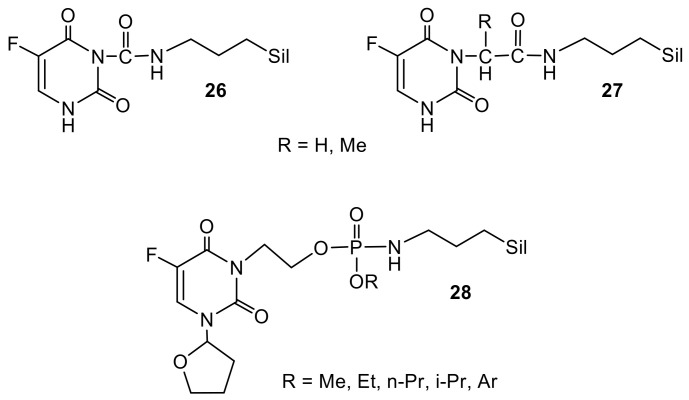
Chemical structures of silatranyl anticancer agents (**26**–**28**).

It is known that 5-fluorouracil (5-FU) and N-1-(2-furanidil)-5-fluorouracil (tegafur) are potent inhibitors of cancer cell growth. At the same time, both 5-FU and tegafur have undesirable side effects and low selectivity of action. To check the synergism between the APS and 5-FU, Ping et al., linked these moieties together (Figure 7) [62]. It was shown that the activity of amide-5-FU derivatives (**26**,**27**) against Hela cells, HT-29 colorectal adenocarcinoma, and MDAMB435 melanoma was quite good and was very close to that of 5-FU (IC_50_ 15 and 10 μg/mL, respectively).

Shi et al. synthesized a series of phosphoramide–tegafur derivatives of 3-aminopropyl-silatrane (**28**) (Figure 7) [63]. The cell toxicity experiments indicated that the compounds (**28**) had an inhibition effect against the adenocarcinoma HCT-8 and hepatocellular carcinoma Bel7402 cell lines (ratios of the inhibition 12–29%).

Thus, silatranes exhibit pronounced antitumor activity both in vitro and in vivo. The dependence of this activity on the structure of silatranes and the substituent at the silicon atom gives hope for the design of effective anticancer drugs.

It is quite obvious that, in the above examples, the molecular hybridization theory (MH) is confirmed in practice (see Refs. [33,34,35,36,37,38,39] and literature cited therein).

## 3. Silatranyl Materials

### 3.1. Silatranyl Chemosensors

**Figure 8:**
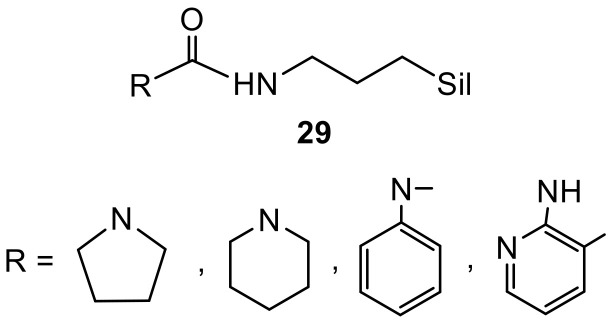
Chemical structures of silatranyl chemosensors (**29**) for the recognition acetate anion.

Chemosensor is a molecular system that can specifically interact with the analyte to produce a detectable signal. The development of silatrane-based chemosensors can lead to the creation of new promising materials [64,65,66,67]. In this line, Singh et al. published a number of papers on the synthesis of hybrid functional derivatives of APS, which are molecular sensors for the recognition of various anions and cations with promising applications [64,65,66,67,68,69]. For example, the fluorescence titration results revealed a selective on–off fluorescence response of urea-silatranes (**29**) toward biologically significant acetate anion (Figure 8) [64].

**Figure 9:**
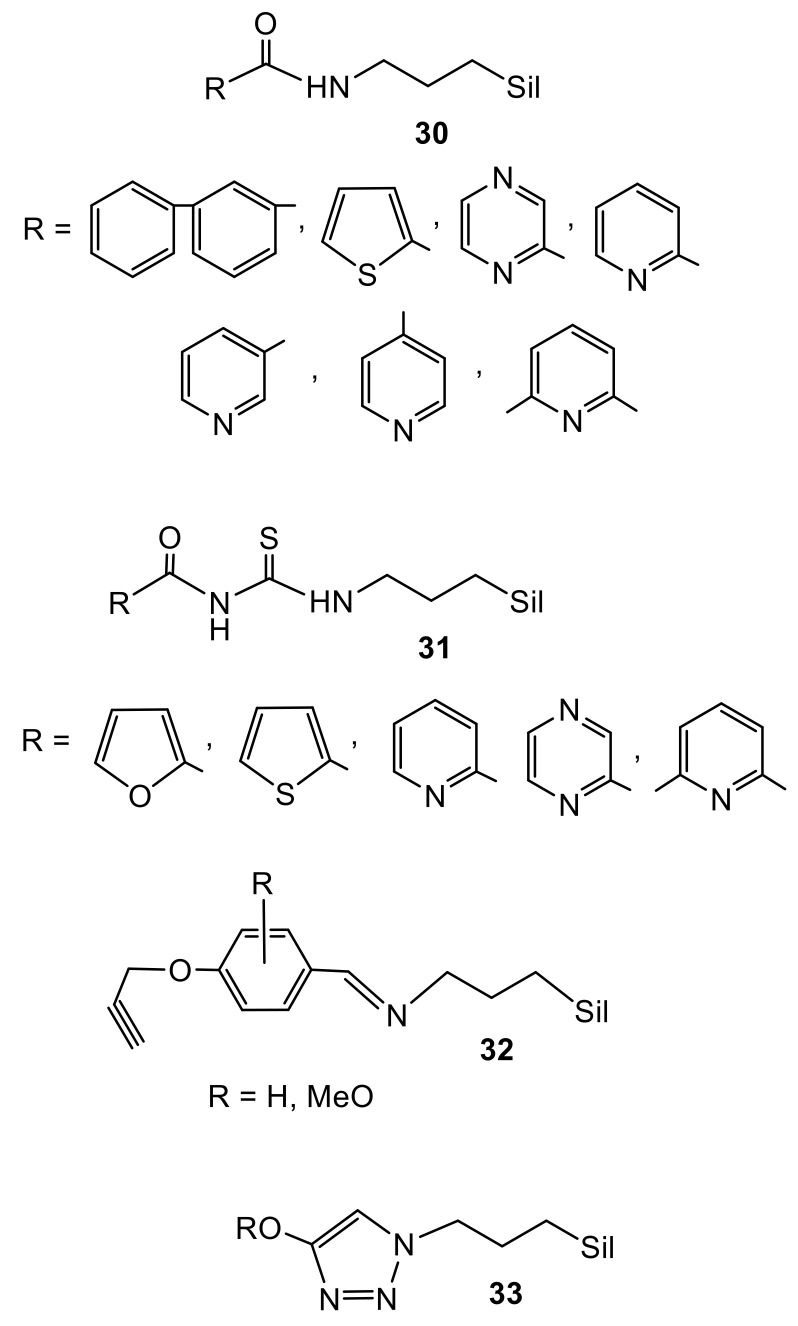
Chemical structures of chemosensors (**30**–**33**) for the detection of metal ions.

Amide-tethered silatranes (**30**) contain Cu^2+^ ion binding sites (Figure 9) [65]. Silatranes with acylthiourea (**31**) have the coordination ability to bind Cu^2+^, Cd^2+^, Hg^2+^,and Pb^2+^ ions (Figure 9) [66]. The UV-visible study of the Schiff-base-acetylene-functionalized organosilatranes (**32**) demonstrated their high selectivity towards the recognition of Zn^2+^ and Co^2+^ ions (Figure 9) [67]. The triazole group has an effective binding unit that can be used for the recognition of various ions. For example, triazolylsilatranes (**33**) showed good selectivity towards metal ions, such as Cu^2+^, Hg^2+^, Pb^2+^ [68], and exceptional selectivity with respect to Ag^+^ (Figure 9) [69].

The recognition and sensing of biologically significant anions has become an important aspect of analytical chemistry. The detection of toxic heavy transition metal cations also becomes the area of prime importance. This is due to the role of these ions in physiology, technology, and environment. The synchronization of silatranyl and other functional moieties provides the effective and promising platform for detection of these ions.

Thus, it is expected that the synthesized chemosensors may offer opportunity for their applications in industry and living sciences.

### 3.2. Silatranyl Dyes and Catalysts

**Figure 10:**
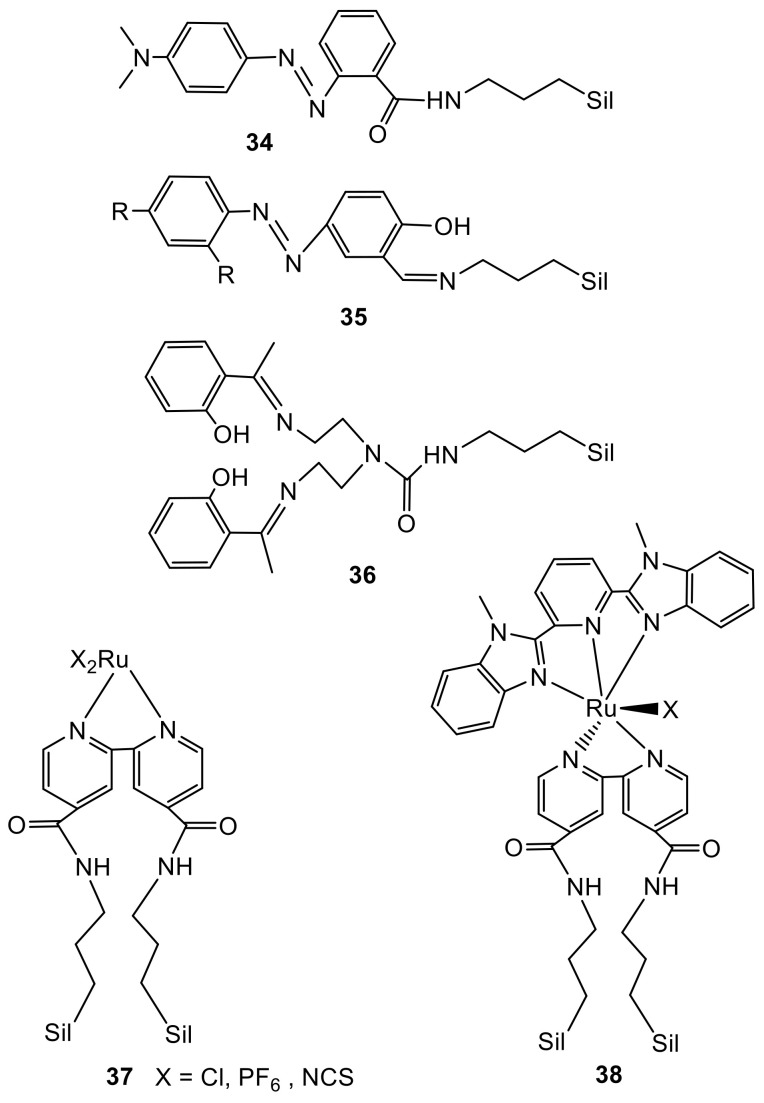
Chemical structures of silatranyl dyes (**34**–**38**).

Natural and synthetic dyes (pigments) are intensively exploited in the paper, textile, food, and other industries. Dyes are fixed on surfaces due to weak non-covalent interactions. In contrast, dyes functionalized with silanes (silatranes) can provide fixation through the formation of strong covalent bonds (see Scheme 3; see Refs. [70,71,72,73] and literature cited therein). At the same time, they possess low toxicity and high stability. In this regard, it was reported [70] on an azo-dye, methyl red, to its silatranyl derivative (**34**) (Figure 10). The covalent fixation of compound (**34**) on the cotton cloth prevented leaching of dye from cloth even after a prolonged treatment with water and soap.

Mutneja et al. obtained and characterized a series of azomethine and diazo-azomethine silatranyl derivatives (**35**–**36**) for immobilization onto silica surface (Figure 10) [71,72,73].

It was shown that the surfaces of silica modified by compounds (**35**) can be successfully used for the capture of copper ions, as well as for the fast uptake of picric acid via the formation of the host–guest complex.

Mesoporous silica with azomethinic pincers (**36**) were employed for the adsorption of anionic dye eriochrome black T and cationic dye methylene blue.

Dye-sensitized solar cells (DSSCs) perform conversion of solar radiation to electricity. Such cells are based on a redox-active chromophore, which can be an organic «dye» or a complex compound of metal. It was reported [74] on the preparation of a number of Ru(II) silatrane-polypyridyl complexes (**37**), which were tested as DSSC «dyes» based on TiO_2_ and WO_3_ (see Scheme 3). It was found that the covalent bond silicon–metal-oxide (-Si-O-M) was more resistant to pH and solvent type than the traditional linkers, thus extending the lifetime of DSSC. Current density (IOC) of (**37**), where R = NCS, on WO_3_ was comparable to the current dye systems.

Later, Materna, Troiano et al. showed that incorporation of the silatrane motif into similar ligands for inorganic complexes (**38**) ensured excellent properties used for fixation of catalysts on metal oxide surfaces (TiO_2_, SnO_2_, nanoITO) under aqueous conditions (Figure 10). For example, silatrane-derived siloxane surface was stable in the range of pH 2 to 11, and the ruthenium complex was found to have stable electrochemical features with repeated cycling [75,76,77,78,79].

It was disclaimed that the silatrane anchors can be bound to metal oxide surfaces at room temperature, and additive of benzoic acid increased the binding by 145%. Besides, it was shown that the released triethanolamine can easily be removed using an aqueous acidic washing or CV cycling [76,77,78,79].

Thus, surface modification with functional silatrane anchors is a promising technique that can find applications in staining, adsorption, catalysis, electronics, semiconductors, biosensor materials, and solar energy conversion devices.

### 3.3. Silatranyl Polymers, Films, Coatings, Materials, and Devices

**Figure 11:**
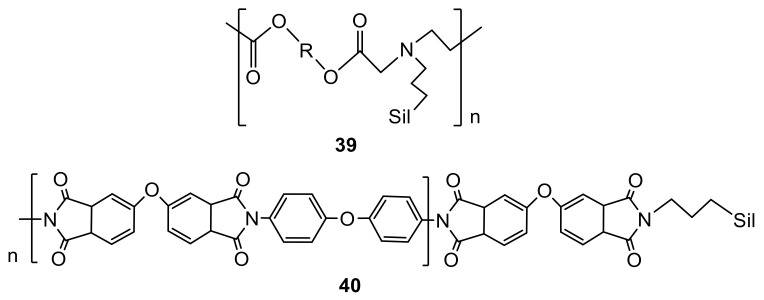
Chemical structures of silatranyl polymers and films (**39**–**40**).

Over the last decade, poly(β-amino esters) have attracted the attention of researchers. They are biodegradable and can be used for the manufacture of medical devices (surgical threads and films, prostheses, implants, grafts, etc.; see Ref. [80] and literature cited therein). Given the beneficial biological activity of silatranes, synthesis of poly(β-amino esters) containing APS is of great practical interest. Such compounds (**39**) were synthesized in the work of Ref. [80] (Figure 11). The obtained polymers possess biological activity characteristic of low molecular APS.

Polyimide (PI) films represent lightweight, flexible polymer-based materials having excellent thermal- and chemical-resistant properties. A series of APS-end-capped PIs (**40**) were prepared by imidization method [81] (Figure 11). The mechanical characteristics and thermal stability of the neat polyimide film increased significantly with 3% loading of APS as end-capping agent. For example, thermal stability increased from 433 to 509 °C, glass transition temperature values enhanced from 240 to 260 °C, tensile strength improved from 118 to 155 MPa, air permeability increased from 0.5464 to 3.2405 cc/s.cm^2^.

Due to their unique properties, nanoparticles (NPs) find wide applications in different fields. For example, noble metal nanoparticles on substrates are often used as special nanomaterials, namely biosensors, catalysts, and amplifiers of electronic and optical signals. In the works of Taiwanese scientists, 3-aminopropylsilatrane (**1**) was employed for anchoring NPs on substrates [82,83,84] of NPs on the silicon surface (SiO_2_) and a good linker for plasmon resonance sensors based on gold nanoparticles (AuNP). In addition, it was revealed that the surfaces treated with APS (**1**) exhibit antifouling properties.

**Figure 12:**
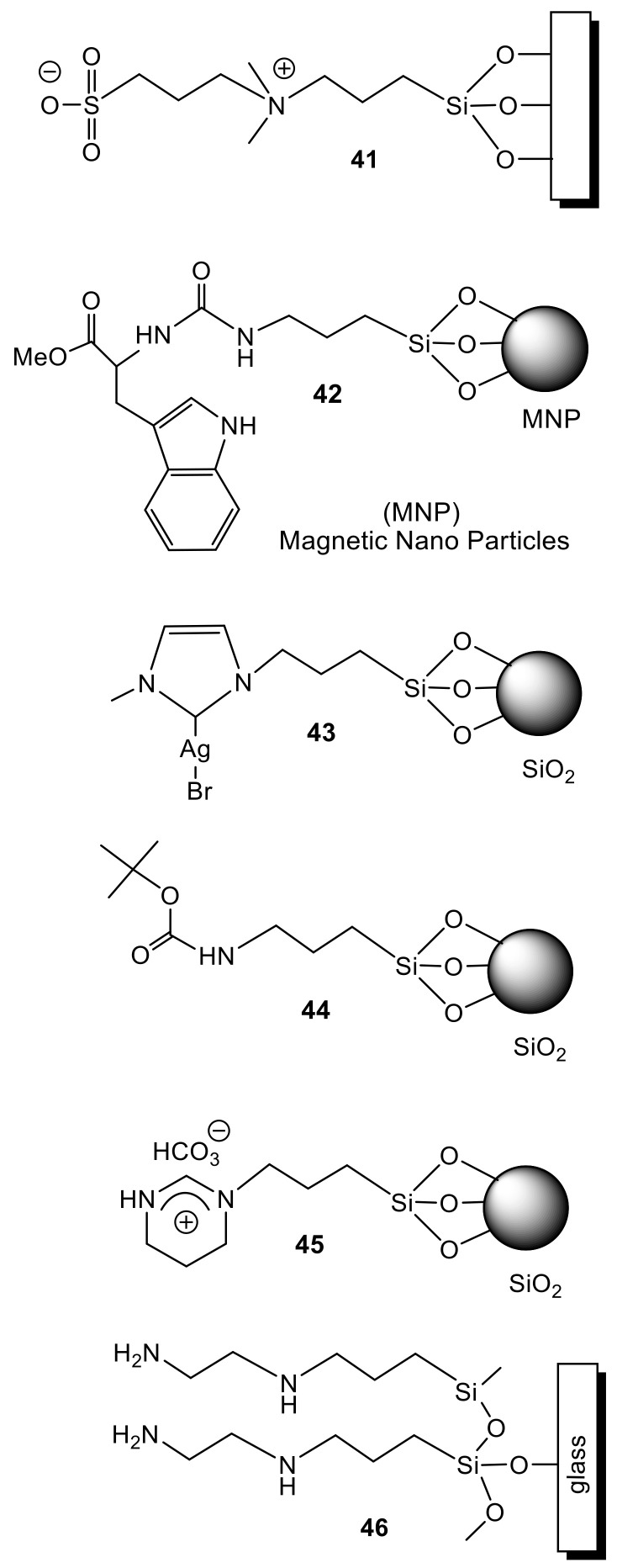
Chemical structures of silatranyl surfaces (**41**–**46**).

Zwitterionic compounds are prospective antifouling materials. A new zwitterionic sulfobetaine silatrane (**41**) (Figure 12) was designed [85]. The excellent antifouling properties of coatings (**41**) were confirmed by their superior resistance to bacterial and protein adsorption (even in complex media, such as human blood and seawater). The fouling levels for all (**41**) coatings are lower than that on bare glass by >99.8%.

A simple and convenient silatrane (**41**)-based filtering system for hemofiltration and collection of circulating tumor cells (CTCs) with fouling resistance and high hemocompatibility and selectivity was developed [86]. It was shown that the proposed device selectively removed the human colorectal carcinoma cell line (HCT116) from circulatory blood. The number of HCT116 cells increased to 10 times more than the initial spiked cell number after incubation for 7 days.

Silatrane-modified surfaces and materials proved to be very useful for visualization and observation of various biological objects. For instance, Lyubchenko et al. described a procedure employing APS (**1**) for successful functionalization of the mica surface [87,88,89]. The material obtained was used for immobilization of DNA, RNA, other biological materials, and their visualizations with atomic force microscopy (AFM). As a result, an instrument capable of imaging the dynamics of molecules at video rate has become available to the biomedical community [88].

Mathur et al., using APS (**1**) as a linker, chemically conjugated tryptophan to the surface of superparamagnetic nanoparticles of iron oxide (Fe_3_O_4_). The synthesized magnetic nanoconjugate (**42**) (Figure 12) was successfully applied for visualization of tumors (cell lines A-549, MCF-7) by scintigraphy, high-resolution transmission electron microscope (HRTEM), and magnetic resonance imaging (MRI). This study showed that the developed nanomagnetic bioconjugate is suitable as a potential diagnostic agent [90].

Garg et al. report the preparation of an Ag(I) complex on silica (**43**) (Figure 12), which appeared to be an efficient heterogeneous catalyst for the regioselective synthesis of triazoles in water at room temperature [91].

A new silatrane with a protected amino group was obtained in Ref. [92]. It was used to design a sensor (**44**) (Figure 12) sensitive to trinitrotoluene (electronic nose) for an ion-sensitive field-effect transistor (ISFET).

Aoyagi and Endo investigated the capture and release of CO_2_ by silatranylamidines in DMSO solution. It was shown that six-membered cyclic amidine with the silatranyl group captured CO_2_ (<99%) at 25 °C, which was released at 60 °C [93]. Currently, on the basis of silatranylamidines, ion-conducting materials (**45**) (Figure 12) are being developed.

The hybrid antifogging materials (**46**) (Figure 12) were obtained in Ref. [94]. They displayed high water absorption (up to 42%), which was 1.5 times higher than that of the typical antifogging polymer polyvinylalcohol (PVA). In addition, these coatings exhibited a scratch resistance four to six times higher than PVA.

Thus, it is shown that silatrane-containing and silatrane-modified hybrid organic-inorganic surfaces and materials (**39**–**46**) demonstrate better properties compared to their individual counterparts.

## 4. Conclusions

Atranes (and their analogues) are chelate organometal/elemental compounds of ethanolamine, diethanolamine, triethanolamine, etc. From a more general point of view, atranes represent a kind of symbiosis of three important components:

(a) Biogenic amino alcohols,
(b) Essential metals/elements: Si, Ge, V, Co, Zn, etc.
(c) Organic substituents

Among atranes, organosilicon derivatives of triethanolamine (silatranes) hold a special place. The unique tricyclic structure (with the Si←N transannular bond) of silatranes imparts them unusual physical and chemical properties. For example, the slow and controlled hydrolysis of silatranes (unlike their predecessors, trialkoxysilanes) allows the self-assembled, stable, morphologically ideal siloxane monolayers on polymeric, metal, glass, textile, and other surfaces to be formed. The high and diverse biological activity of silatranes is ensured by the mutual influence of the silatrane cell and the substituent at the silicon atom.

Thus, the combination of silatranes (for example, cheap and available 3-aminopropylsilatrane) with functional or pharmacophoric units in one molecule permits to synthesize original silicon-containing hybrid compounds. The latter can be successfully employed for the design of efficient physiologically active substances (plant growth regulators, antifungal, antimicrobial, antiparasitic, anticancer agents), as well as medical, optical, catalytic, sorption, and special polymeric materials and products (chemosensors for the recognition of anions and detection of metal ions, special dyes, as well as silatranyl polymers, films, coatings, materials, and devices) with improved properties.

In the present review, it is highlighted that the development of hybrid silatranes could have a significant impact on chemical, biological, pharmaceutical, agricultural, environmental, and materials science research.

## Data Availability

Not applicable.
